# Genetic deletion of HVEM in a leukemia B cell line promotes a preferential increase of PD-1^-^ stem cell-like T cells over PD-1^+^ T cells curbing tumor progression

**DOI:** 10.3389/fimmu.2023.1113858

**Published:** 2023-03-23

**Authors:** Maria-Luisa del Rio, Carla Yago-Diez de Juan, Giovanna Roncador, Eduardo Caleiras, Ramón Álvarez-Esteban, José Antonio Pérez-Simón, Jose-Ignacio Rodriguez-Barbosa

**Affiliations:** ^1^ Transplantation Immunobiology and Immunotherapy Section, Institute of Molecular Biology, University of Leon, Leon, Spain; ^2^ Monoclonal Antibodies Unit, National Center for Cancer Research (CNIO), Madrid, Spain; ^3^ Histopathology Core Unit, National Center for Cancer Research (CNIO), Madrid, Spain; ^4^ Section of Statistics and Operational Research, Department of Economy and Statistics, University of Leon, Leon, Spain; ^5^ Department of Hematology, University Hospital Virgen del Rocio / Institute of Biomedicine (IBIS / CSIC), Sevilla, Spain

**Keywords:** HVEM: herpesvirus entry mediator, BTLA: B- and T-lymphocyte attenuator, PD-1: programmed death 1, Tim 3: T-cell immunoglobulin and mucin-domain containing-3, hybrid resistance

## Abstract

**Introduction:**

A high frequency of mutations affecting the gene encoding Herpes Virus Entry Mediator (HVEM, TNFRSF14) is a common clinical finding in a wide variety of human tumors, including those of hematological origin.

**Methods:**

We have addressed how HVEM expression on A20 leukemia cells influences tumor survival and its involvement in the modulation of the anti-tumor immune responses in a parental into F1 mouse tumor model of hybrid resistance by knocking-out HVEM expression. HVEM WT or HVEM KO leukemia cells were then injected intravenously into semiallogeneic F1 recipients and the extent of tumor dissemination was evaluated.

**Results:**

The loss of HVEM expression on A20 leukemia cells led to a significant increase of lymphoid and myeloid tumor cell infiltration curbing tumor progression. NK cells and to a lesser extent NKT cells and monocytes were the predominant innate populations contributing to the global increase of immune infiltrates in HVEM KO tumors compared to that present in HVEM KO tumors. In the overall increase of the adaptive T cell immune infiltrates, the stem cell-like PD-1^-^ T cells progenitors and the effector T cell populations derived from them were more prominently present than terminally differentiated PD-1^+^ T cells.

**Conclusions:**

These results suggest that the PD-1^-^ T cell subpopulation is likely to be a more relevant contributor to tumor rejection than the PD-1^+^ T cell subpopulation. These findings highlight the role of co-inhibitory signals delivered by HVEM upon engagement of BTLA on T cells and NK cells, placing HVEM/BTLA interaction in the spotlight as a novel immune checkpoint for the reinforcement of the anti-tumor responses in malignancies of hematopoietic origin.

## Introduction

Herpesvirus entry mediator (HVEM) is a tumor necrosis factor receptor superfamily (TNFRSF) member 14 (TNFRSF14) that interacts with two members of the immunoglobulin superfamily, BTLA and CD160, and a member of the TNFSF (TNFSF14, LIGHT, homologous to lymphotoxin, exhibits inducible expression and competes with HSV glycoprotein D for binding to herpesvirus entry mediator, a receptor expressed on T lymphocytes). As opposed to PD-L1 that exhibits a hematopoietic restricted pattern of expression, HVEM is expressed on both hematopoietic and non-hematopoietic cells. Consequently, it may regulate both T cell priming in the draining lymph node and the effector phase of the T cell response at the inflamed tissue site by delivering a balance of negative signals upon BTLA engagement and positive signals upon interaction with LIGHT or CD160 (1, 2). Besides, the engagement of HVEM on T cells by its ligands stimulates T cell survival (3–6). Despite the fact that HVEM potentially delivers two opposing signals, co-inhibition is predominant over co-stimulation as revealed the phenotype of HVEM and BTLA deficient mice, which are more susceptible than WT to develop experimentally induced encephalitis (1, 7). Apart from BTLA, CD160 may also play a minor role as co-inhibitory receptor in a small subpopulation of human CD4 T cells (8) and in NKT cells (9). Despite these findings, the current view is that CD160 in NK cells as well as in CD8 T cells functions as a costimulatory receptor (10–13).

We hypothesized that HVEM expression on hematopoietic tumor cells may serve as a cell extrinsic factor that can modulate the anti-tumor response by co-inhibiting the function of T cells and NK cells through BTLA. To assess the role of the HVEM expression on tumor implantation and the subsequent dissemination, we evaluated the impact of this molecule in the modulation of the anti-tumor response. To that aim, the gene encoding HVEM was genetically inactivated. Then, HVEM WT or HVEM KO tumor cells were implanted into F1 recipients, in which NK cells and T cells participate in tumor rejection, and tumor engraftment was evaluated in spleen and bone marrow, whereas tumor immune infiltrates were analyzed in the metastatic nodules of the liver.

Our findings support the notion that HVEM expression on tumor cells provides them with a survival advantage to resist the anti-tumor response mediated by NK cells and T cells putting HVEM in the spotlight of a potential therapeutic target for immune intervention in tumors of hematological origin.

## Material and methods

### Mice

Eight to twelve-week old F1 hybrid female mice (♀Balb/c AnN × ♂C57BL/6J) (H-2^d/b^) were generated by crossing the parental mouse lines in our animal facility (University of Leon, Spain). All animal experiments have been approved by the Animal Welfare Committee of the University of Alcala de Henares (Madrid) and handled in accordance with the European Guidelines for Animal Care and Use of Laboratory Animals (authorization # OH-UAH-2016/015).

### A20 lymphoma cell line

The A20 transplantable leukemia cell line was derived from B lymphocytes of a naturally occurring reticulum cell sarcoma from an old Balb/c AnN mouse (H-2^d^, TIB-208, ATCC, American Type Culture Collection, Manassas, VA, USA) (14, 15). This B cell malignancy was later classified as diffuse large B cell lymphoma because it was found to be originated from mature B cells of the germinal center (14). For the *in vitro* culture, A20 tumor cells and its derivatives were grown in complete RPMI-1640 medium (Sigma-Aldrich, St. Louis, MO) supplemented with 10% fetal calf serum (Hyclone), 2 mM L-glutamine (Sigma), 1 mM Pyruvate, (Sigma), 1× non-essential amino acids and 0.05 mM 2-mercaptoethanol (Sigma-Aldrich, St. Louis, MO) at 37 °C and 5% CO_2_.

### Generation of GFP transgenic A20 leukemia cells

In order to discriminate A20 leukemia cells (B cell lineage) from host B cells and the rest of immune cells of the recipient F1 mice, parental A20 tumor cells were transduced with a lentiviral vector encoding the enhanced green fluorescent protein (EGFP) (pLJM1-EGFP). pLJM1-EGFP was purchased from Addgene (Dr. David Sabatini, plasmid # 19319) (16). A third-generation packaging system was used to produce viral particles in HEK293T cells. Three days after the infection, cells were cloned by limiting dilution and then screened for the selection of clones expressing green fluorescence protein. A stable clone of A20 HVEM WT tumor cells expressing EGFP (clone named LCL-7) was chosen for further studies and from this cell line, HVEM deficient A20 leukemia cells were derived (clone LCL-25) that were routinely tested by PCR to rule out the presence of mycoplasma contamination (17).

### CRISPR/Cas9-mediated generation of HVEM deficient A20 leukemia cells

The coding sequence of mouse TNFRSF14 (HVEM) was retrieved from GenBank accession number (NM_178931.2) and ensembl database (ENSMUSG00000042333) to design the targeting strategy. The gene encoding HVEM is composed of eight exons, where exon 1 and the first part of exon 2 together encode the leader peptide sequence. The second part of exon 2 and exon 3 to exon 6 correspond to cysteine rich domains (CRD) 1-4 and the final part of exon 6 to exon 8 encode the transmembrane (TM) and intracellular regions (IC) (**Supplementary Figure 2A**).

CRISPR/Cas9 (Clustered, regularly interspaced, short palindromic repeats–associated nuclease Cas9) approach was applied to target HVEM expression in A20 cell line (18, 19). Four different sgRNA guides targeting exon 1 were selected from the predicted output of potential oligonucleotide sequences with good on-target score in chopchop web site: https://chopchop.cbu.uib.no/, then tested in an *in vitro* assay for assessing their cutting efficiency and sgRNA-mHVEM-1 (+) was chosen for gene inactivation. Indel mutational activity of each targeting vector was determined using the T7EI mismatch detection assay (13, 20) (**Supplementary Figure 2B**). When necessary, a G nucleotide at the start of the primer sequence for U6 transcription was added and the primer sequences were then flanked in both ends by *BsmBI* restriction sites (**Table 1**). The Lenti-CRISPR-v2 plasmid encoding Cas9 and including a puromycin cassette (Dr. Feng Zhang, Addgene plasmid # 52961) was used to clone an oligo DNA guide designed to target exon 1 of the gene encoding HVEM (**Table 1**) (21, 22). Then, each set of forward and reverse sgDNA oligonucleotides was annealed, phosphorylated and cloned into a previously dephosphorylated destination vector lentiCRISPR v2. The plasmid construct lentiCRISPR v2 containing sgRNA-mHVEM-1 (+) oligowas then electroporated into A20-GFP leukemia cells (clone LCL-7). The bulk of A20 electroporated cells were cloned by limiting dilution in the presence of puromycin and screened by flow cytometry with a rat anti-mouse HVEM monoclonal antibody (clone 6C9) to select a A20 cell clone lacking HVEM expression that was termed LCL-25 (6). The mutation introduced into HVEM gene was further amplified by PCR using flanked primers located at both sides of the targeted exon 1 that spanned from the 5´UTR sequence located upstream of the start codon to intron 1-2 (HVEM-mut-F2: 5´GGAGGTAGGTCAAAAGATC3´ and HVEM-mut-RC2: 5´GCATGCTCTGCTTCTTGAA 3´) and then subjected to sequencing for further characterization.

**Table 1:** Oligo sgRNA guides for targeting exon 1 encoding the initial part of the signal peptide.

sgRNA guide name	Target exon	Target site without NGG flanked by *BsmbI* restriction sites (add G, if necessary) 5´→ 3´
**mHVEM-1 (+)**	**Exon 1**	**G-CAGGATGGGGGTCGGCACCCTGG**
**mHVEM-3 (-)**	**Exon 1**	**G-CCAGGATGGGGGTCGGCACCCTG**
**mHVEM-6 (-)**	**Exon 1**	**G-CCCTACAGACAACACCTTCAGGC**
**mHVEM-13 (+)**	**Exon 1**	**G-TGGAACCTCTCCCAGGATGGGGG**

### Mouse model of intravenous parental tumor implantation into non-irradiated F1 recipients

A20 parental leukemia cells evade the anti-tumor response mediated by hybrid resistance mechanisms of rejection as long as a sufficient number of tumor cells are injected to overcome the initial NK cell-mediated immune barrier known as hybrid resistance (23, 24). HVEM WT and HVEM KO leukemia cells were grown and expanded in complete RPMI culture medium at a cell density of 3 × 10^5^ cells/ml, collected at logarithmic phase of cell growth, washed twice with D-PBS and 5 × 10^6^ cells were injected intravenously into F1 recipients in a volume of 200 microliters (23). One month after the adoptive transfer of the tumor cells, F1 recipient mice were euthanized by CO_2_ inhalation and the extent of tumor dissemination was evaluated in two hematopoietic compartments (spleen and bone marrow) and also in the metastatic nodules of the liver.

### Flow cytometry analysis of tumor infiltrating leukocytes

The distinction of tumor cells from non-tumor cells infiltrating metastatic nodules of the liver, spleen and bone marrow was possible thanks to the transduction of tumor cells with green fluorescence gene (EGFP). The phenotypic characterization of tumor infiltrating leukocytes (TILs) invading the metastatic nodules of the liver was carried out by flow cytometry using the set of monoclonal antibodies listed in **Table 2**, paying particular attention to cell markers that permits the characterization of the T cell heterogeneity present in the immune infiltrates of the metastatic tissue. The phenotypic characterization of the heterogeneous populations of CD4 T cells and CD8 T cells infiltrating the tumors was approached following a linear scheme of T cell differentiation put forward by Anderson et al. for CD8 T cells has been illustrated in **Supplementary Figure 1** (27, 28).

**Table 2:** List of antibodies: specificity, biotin/fluorochrome labeling, clone name and the provider.

Receptor	Labeling	Clone	Company
Lineage restricted cell surface markers			
Isotype rat IgG_2a_	Bio	AFRC MAC 157	Home-made (ECACC)
MHC-I (H-2K^b^)	APC-eFluor 780	AF6-88.5.5.3	Thermofisher, #47-5958-82
MHC-I (H-2K^d^)	FITC	SF1-1.1	Biolegend, #116606
CD11b	PerCP-Cy5.5	M1/70	BD, #561114
CD3	BV711	17A2	Biolegend, (#100241)
CD4	PE-Cy7	GK1.5	Biolegend, # 100422
CD8α	PE	53-6.7	Biolegend, # 100708
CD8α	BV510	53-6.7	Biolegend, #100752
Ly6C	Bio	Monts-1	Home made (25)
Ly6G	PE	1A8	BD, # 551461
CD49b	APC	DX5	Biolegend, #108910
HVEM/BTLA/CD160 pathway			
BTLA	Bio	4G12b	Home-made (26)
CD160	Bio	6H8	Home-made (13)
HVEM	Bio	6C9	Home-made (6)
T cell differentiation molecules			
KLRG-1 (MAFA)	BV785	2F1/KLRG	Biolegend, # 138429
IL-7Rα	PE	SB/199	Biolegend, #121111
CD44	PE-Cy7	IM7	Biolegend, # 103029
CD62L	AF647	Mel-14	Biolegend, 104421
CD137 (4-1-BBL)	Bio	17B5	Thermofisher, #13-1371-82
CD27	Bio	L6.3A10	Biolegend, # 124205
ICOS	Bio	C398.4A	Thermofisher, #13-9949-82
Co-inhibitory molecules			
TIGIT	PE-Dazzle 594	1G9	Biolegend, #142109
VISTA (PD-1H)	PE	MIH64	BD, # 566269
PD-1	BV421	29F.1A12	Biolegend, #135217
Tim-3	Bio	8B.2C12	Thermofisher, # 13-5871-85
CD319 (SLAMF7)	PE	4G2	Biolegend, # 152005
CD39	PE-Cy7	Duha59	Biolegend, # 143805
Transcription factor and cell membrane surrogate			
Ly108 (SLAMF6)	PE	330-AJ	Biolegend, #134605
TCF-7/TCF-1	AF647	S33-966	BD, #566693
Chemokine receptors			
CX_3_CR1	AF700	SA011F11	Biolegend, # 149035
CCR3 (CD193)	PE	83101	R&D systems, #FAB729P
CCR5 (CD195)	Bio	C34-3448	BD, #559922
CCR7 (CD197)	Bio	4B12	Biolegend, #120103
CCR9 (CD199)	PE	CW-1.2	BD, #565576
CXCR3 (CD183)	Bio	CXCR3-173	Thermofisher, #13-1831-82
CXCR4 (CD184)	BV421	L276F12	Biolegend, # 146511
CXCR5 (CD185)	PE	L2138D7	Biolegend, # 145503

ECACC, European Collection of Authenticated Cell Cultures.

All the antibodies used in this work were purchased from Biolegend, Thermofisher or Beckton Dickinson or were produced, labeled and titrated in house from our own stocks. Fc receptors were blocked with unlabeled anti-FcγR mAb (2.4G2) by incubating cell suspensions with 0.2 μg of antibody per 1×10^6^ cells to reduce non-specific binding before adding the cocktail of antibodies for each staining. The binding of biotinylated antibodies to cell surface and intracellular antigens was detected with either streptavidin (SA)-PE, SA-PECy7 or SA-BV421, depending on the combination of fluorochromes used in each particular staining. Dead cells and debris were systematically excluded from the acquisition gate by adding propidium iodide (PI) prior to data acquisition. Living cells were gated as PI negative and cellular aggregates were excluded according to FSC-H /FSC-A dot plot profile. For the detection of transcription factor TCF-1 expression in T cells infiltrating the tumor, cells were first incubated with a fixable viability dye (Zombie Violet, Biolegend) to exclude dead cells from the gate of analysis followed by staining of surface markers using the fixation/permeabilization Foxp3/Transcription Factor Staining Buffer Set from Thermofisher (#00-5523). Flow cytometry acquisition was conducted on a Cytek® Aurora Spectral Cytometer with four lasers and data analysis was performed using FlowJo software version 10.

### Immunohistochemistry

Tissue samples were fixed in 10% neutral buffered formalin (4% formaldehyde solution), embedded in paraffin and cut at 3 µm, mounted on superfrost®plus slides and dried overnight. Immunohistochemistry reactions were performed on an automated immunostaining platform (Autostainer Link 48, Dako) for CD8 and (Discovery XT-ULTRA, Ventana-Roche). For CD8 staining, antigen retrieval was performed with high pH buffer, PT Link Dako, Agilent and endogenous peroxidase was blocked (3% hydrogen peroxide). The slides were then incubated with rat monoclonal, anti-CD8a primary antibody (clone OTO94A1/200 30min, Monoclonal Antibodies Core Unit). Horseradish peroxidase-conjugated anti-rat secondary antibody (biotinylated rabbit anti Rat Vector BA-4001). After the primary antibody, slides were incubated with the corresponding secondary antibodies (rabbit anti-rat, Vector (BA-4001) and visualization systems (OmniRabbit, Ventana, Roche). For PD-1 staining, antigen retrieval was first performed with the appropriate pH buffer (CC1 Ventana, Roche), and endogenous peroxidase was blocked (peroxide hydrogen at 3%). Then, slides were incubated with Rabbit Monoclonal anti- PD-1 (clone D7D5W, 1/50 40min, Cell Signaling, 84651). After the primary antibody, slides were incubated with visualization systems (OmniMap anti-Rabbit, 760-4311, Ventana, Roche).

Whole digital slides were acquired with a slide scanner (Axio Scan Z1, Zeiss), and images captured with the Zen Blue Software (Zeiss). Digital image analysis were performed with Qupath 0.3.0 (29). We used watershed positive cell detection with the following settings: Detection image: Optical density sum; requested pixel size: 0.5 µm; background radius: 20 µm; median filter radius: 0 µm; sigma: 1.8 µm; minimum cell area: 10 µm2; maximum cell area: 400 µm2; threshold: 0.1; maximum background intensity: 2 and cell: DAB OD mean:0.2.

### Statistical analysis

Sample normality distribution were tested by using D´Agostino and Pearson tests. When samples followed a normal distribution, the parametric unpaired Student´s *t* test was chosen for the evaluation of the existence of significant differences between means. Otherwise, the non-parametric Mann-Whitney statistical test was applied to compare the differences of medians. The statistical analysis was performed using GraphPad Prism 7.0 software (GraphPad Software, Inc). The criterion for the establishment of statistical significance were as follows: *p* < 0.05 (*), p < 0.005 (**) and p < 0.0005 (***) and NS (non-significant).

## Results

### CRISPR/Cas9 gene inactivation of HVEM expression in A20 leukemia cells

HVEM is a molecular hub that exchanges bidirectional information through different circuits upon interaction with its main high affinity partners BTLA/CD160/LIGHT and the weak affinity partner LTα (30–32). To gain insight into the role of HVEM/BTLA/CD160/LIGHT pathway in the net balance of co-inhibitory and costimulatory signals provided by tumor cells to T cells and NK cells, HVEM gene was mutated in A20 leukemia cells and the anti-tumor response was evaluated in the context of hybrid resistance and parental tumor implantation into F1 recipients.

By using a CRISPR/Cas9 approach, the gene encoding HVEM was targeted at exon 1 to introduce indel mutations that led to knocking out the sequence encoding the signal peptide and thus preventing protein expression on the cell surface of tumor cells (19, 22). The PCR amplicon yielded a band of 1090 bp for the WT cell line and a band of 618 bp for the KO cell line (a deleted fragment of 472 bp spanned upstream 5`UTR, 5`UTR region sequence, exon 1 and part of intron 1-2 sequences) (**Supplementary Figure 2C**). The amino acid sequence alignment of HVEM WT versus KO shows that exon 1 deletion led to elimination of the first part of the signal peptide (**Supplementary Figure 2D**). The absence of protein expression was then evidenced by flow cytometry using an anti-HVEM monoclonal antibody (clone 6C9), previously characterized in our laboratory (6) (**Supplementary Figure 2E**). As described in many other hematological tumors such as diffuse large B cell lymphoma, follicular lymphoma and Burkitt lymphoma, all of them classified as non-Hodgkin lymphomas that stem from germinal center B cells, all express low levels of HVEM (33). This claim also applies to A20 leukemia cells, in which the level of HVEM expression was confirmed to be low, whereas its binding partner BTLA receptor was strongly expressed (**Supplementary Figure 2E**). HVEM expression seemed to be necessary for maximal expression of BTLA, because in the absence of HVEM, the fluorescence intensity level of BTLA was slightly decreased (**Supplementary Figure 2E**)

Altogether, the data presented herein shows that the indel mutation introduced into exon 1 encoding the initial part of the signal peptide led to successful inactivation of HVEM protein expression on the cell membrane of A20 leukemia cells.

### HVEM WT and HVEM KO tumor cells exhibited a similar *in vitro* proliferation rate

The *in vitro* rate of cell proliferation and cell death reflects the accumulation of cells surviving after each cycle of division. To quantify the number of cells surviving in cell culture and determine the impact of HVEM deficiency on their overall survival, an equal number of HVEM WT or HVEM deficient tumor cells were seeded in 24 well plates under the same culture conditions and cell counting was performed every day from day 1 to day 6. **Supplementary Figure 2F** shows that both cell lines followed a similar and parallel growth rate regardless of whether they were HVEM WT or HVEM deficient.

The proliferation data indicates that the abrogation of HVEM expression in A20 leukemia cell line did not compromise its *in vitro* replication rate.

### HVEM deficiency impaired tumor colonization of spleen, bone marrow and metastatic nodules of the liver

Transplantable hematopoietic tumors of B cell origin injected intravenously disseminate throughout the blood stream rapidly colonizing the bone marrow niche and the secondary lymphoid organs, spleen and peripheral lymph nodes where mature B cells reside. The extent of parental tumor implantation in these two compartments of F1 recipients is dependent on the local immune hybrid resistance mechanisms that hamper tumor engraftment.

**Figure 1:**
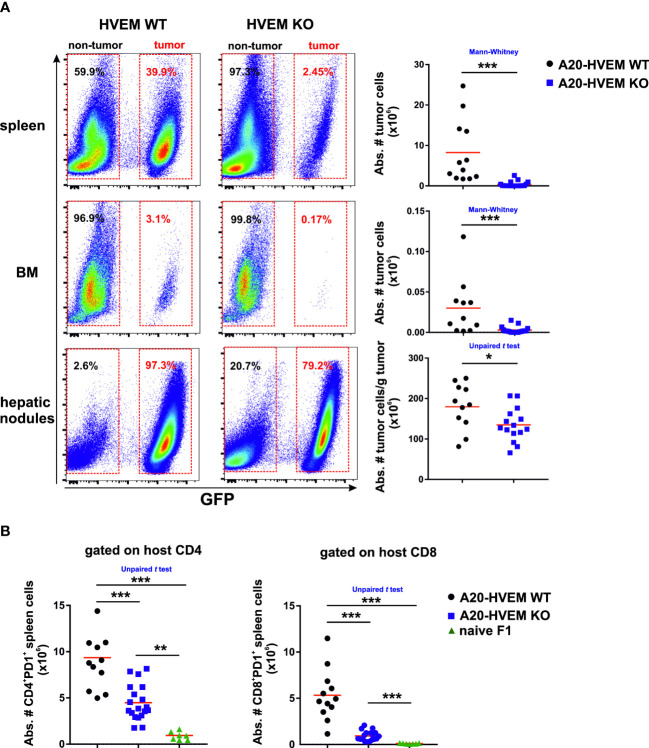
HVEM WT leukemia cells engrafted more aggressively in primary and secondary lymphoid compartments than HVEM-deficient leukemia cells in a parental tumor mouse model implanted into F1 recipients. 5×10^6^ LCL-7 (HVEM WT) or LCL-25 (HVEM KO) tumor cells were intravenously inoculated in F1 mice. Thirty days after tumor implantation, mice were euthanized and the engraftment of WT and KO tumor cells in spleen (**A**, upper panel), bone marrow (**A**, middle panel) and metastatic hepatic nodules (**A**, lower panel) was analyzed based on the expression of GFP. The absolute number of WT and KO tumor cells was calculated in each compartment. **(B)** The absolute number of host CD4 T cells (**B**, left panel) and CD8 T cells (**B**, right panel) expressing PD-1 was evaluated in spleens of naïve F1 mice (control) as well as in F1 recipients inoculated with either HVEM WT or HVEM KO tumor cells. Data show the mean from 10-17 mice per group pooled from two independent experiments. Statistical analyses were performed using two-tailed non-parametric Mann–Whitney U test or two-tailed unpaired Student´s *t* test. *, p<0.05, **, p<0.005 and ***, p<0.0005.

We found that HVEM expression on tumor cells was required for tumor colonization of spleen and bone marrow and metastatic nodules of the liver. The absence of HVEM expression on tumor cells significantly impaired tumor colonization of spleen and bone marrow compared to that observed in HVEM WT tumor cells, being the bone marrow particularly resistant to tumor engraftment (**Figure 1A**, upper, middle and lower panel).

The loss of HVEM expression on tumor cells worsen their fitness to survive in the host likely due to an increased vulnerability to the overall innate and adaptive anti-tumor immune response.

### Tumor burden in spleen of F1 recipients correlated with an increase of CD4 and CD8 T cell numbers co-expressing PD-1

The spleen is a secondary lymphoid organ rich in immune cells involved in hybrid resistance.There was a significantly greater number of host CD4 T cells and CD8 T cells expressing PD-1 in spleens of F1 mice receiving HVEM WT leukemia cells than those receiving HVEM KO leukemia cells (**Figure 1B**).

In brief, the cell counts of T cells co-expressing PD-1 in the spleen was proportional to the tumor mass.

### Hepatic tumor burden owing to nodular metastases was significant larger in F1 recipients of HVEM WT tumor cells than in those receiving HVEM deficient tumor cells

**Figure 2:**
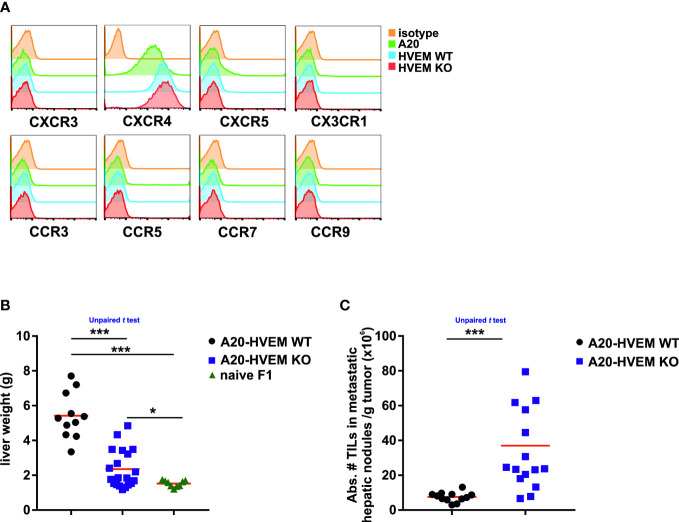
HVEM-expressing tumor cells colonize the liver of F1 recipients more efficiently than their HVEM-deficient counterparts. One month after the intravenous injection of LCL-7 or LCL-25 cell lines into F1 recipients, tumor metastases of the liver were isolated and tumor weight and T cell infiltration was assessed. **(A)** The expression profile of chemokine receptors (CXCR3, CXCR4, CXCR5, CX3CR1, CCR3, CCR5, CCR7 and CCR9) was evaluated in A20, A20-HVEM WT and A20 HVEM KO tumor cell lines. The livers of F1 mice receiving either HVEM WT or HVEM KO tumor cells were weighted and compared to that of F1 naïve mice **(B)** and the number of tumor infiltrating leukocytes (TILs) was calculated **(C)**. Data show the mean from 12-16 mice per group from two independent experiments. Statistical analyses were performed by unpaired Student’s two-tailed *t* test. *, p<0.05, ***, p<0.0005.

Mature B cells express the chemokine receptor CXCR4, which is essential for the process of tumor lymphomagenesis, infiltration, and retention of leukemia cells in the metastatic sites (34). To assess the relevance of these previous findings in human diffuse large B cell lymphomas, the phenotypic pattern of chemokine receptor expression was analyzed in A20 HVEM WT and HVEM KO leukemia cells with a set of antibodies against different chemokine receptors (CXCR3, CXCR4, CXCR5, CX3CR1, CCR3, CCR5, CCR7 and CCR9). Of all, only one of them, CXCR4 stood out and turned out to be positive and highly expressed in the parental A20 cell line and its derivatives, whereas expression of CCR7 or CXCR5 was undetectable (**Figure 2A**).

The increase in size of the liver due to accumulation of tumor metastases was significantly greater in F1 recipients of HVEM WT tumor cells than in those receiving HVEM KO leukemia cells (**Figure 2B**, left panel). A striking observation was that the less tumor burden in the liver, the more abundant was the infiltration of immune cells per gram of tumor. Thus, a significant much greater number of immune cells infiltrates were found in tumors originated from HVEM deficient leukemia cells than those originated from HVEM WT leukemia cells (**Figure 2C**, right panel).

The loss of HVEM in leukemia cells was associated with an increased abundance of immune infiltrates suggesting that HVEM expression confers protection against the immune rejection.

### Innate immune cells were significantly augmented in infiltrates of HVEM deficient tumors

A common consensus agreement among pathologists is that massive infiltration of immune cells in tumors (hot tumors) correlates with good prognosis in patients suffering from hematological and non-hematological malignancies (35). Innate NK cells are the major effector immune cells involved in the rejection of parental bone marrow cells or parental tumors of hematopoietic origin transplanted into F1 recipients (36, 37). Apart from these innate lymphoid cells, myeloid cells are also often present at later stages of tumor progression behaving as immunosuppressive cells that are capable to condition the tumor microenvironment to favor tumor growth (38, 39).

**Figure 3:**
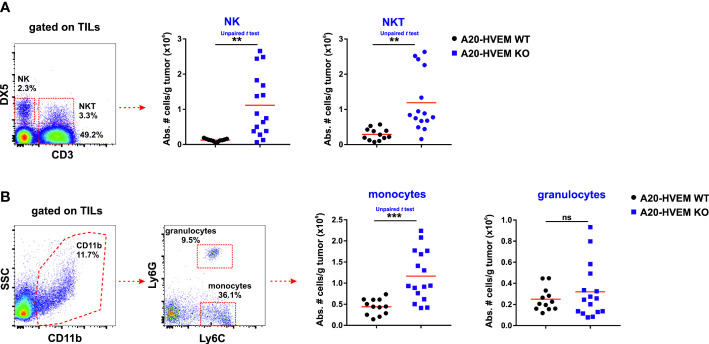
Innate tumor leukocyte infiltrating cells are significantly more abundant in mice injected with tumor cells devoid of HVEM. Hepatic metastatic nodules were isolated from F1 mice injected with either HVEM WT or HVEM-deficient tumors and the absolute numbers of NK cells and NKT cells **(A)** as well as monocytes and granulocytes **(B)** was determined. Representative dot plots depicting the gating strategy and the subpopulations of each analysis are shown. Data indicate the mean from 12-16 mice per group from two independent experiments. Statistical analysis was calculated using two-tailed unpaired Student‘s *t* test. **, p<0.005 and ***, p<0.0005. ns, non-significant.

We took advantage of the metastatic behavior of A20 leukemia cells for the liver parenchyma and used the metastatic hepatic nodules for the evaluation of immune infiltrates invading HVEM WT and HVEM KO tumors (40, 41). The innate immune infiltrates identified within the tumor stroma were bone marrow-derived NK cells and thymus-derived NKT cells, both stemming from lymphoid precursors. In the metastatic nodules of the liver, the innate immune infiltrates were significantly more abundant in TILs isolated from HVEM KO tumors than in those isolated from HVEM WT tumors. Thus, the absolute number of NK cells (CD3^-^ DX5^+^), NKT cells (CD3^+^ DX5^+^) (**Figure 3A**) and monocytes (CD11b^+^ / Ly6C^+^ / Ly6G^-^) (**Figure 3B**) infiltrating the tumors was significantly much more abundant in immune infiltrates of HVEM deficient tumors than in HVEM WT tumors. However, granulocytes (CD11b^+^ / Ly6C^+^ / Ly6G^+^) infiltrating HVEM WT or HVEM KO tumors did not reach a statistically significant difference (**Figure 3B**). A 10-fold and 4-fold increase of NK cells and NKT cells was observed in HVEM KO tumors compared to HVEM WT tumors, respectively, whereas the contribution of monocytes to the global increase was minor and no changes were observed in the granulocyte population.

In summary, NK cells and NKT cells are the major contributors of the innate immune response that account for the reduced size of metastatic lesions of the liver in HVEM KO tumors compared to their WT counterparts.

### Significant and preferential increase of PD-1^-^ over PD-1^+^ T cell immune infiltrates in liver metastases of HVEM deficient tumors

Apart from NK cells, which are the dominant mediators in hybrid resistance against parental tumors in the initial phase of tumor implantation, CD4 T cells and CD8 T cells can also recognize tumor-specific antigens in the subsequent stages of tumor progression. Host APC capture these tumor antigens to be presented to T cells and thus promote their differentiation towards effector T cells, resisting tumor engraftment. CD4 T cell help is required to support CD8 T cell responses through licensing of DCs that increase antigen presentation and co-stimulatory signals when CD4 T cells and CD8 T cells are recognizing foreign peptide/MHC complexes on the same DC (42, 43). CD4 T cells also contribute to the cytotoxic anti-tumor response (44).

**Figure 4:**
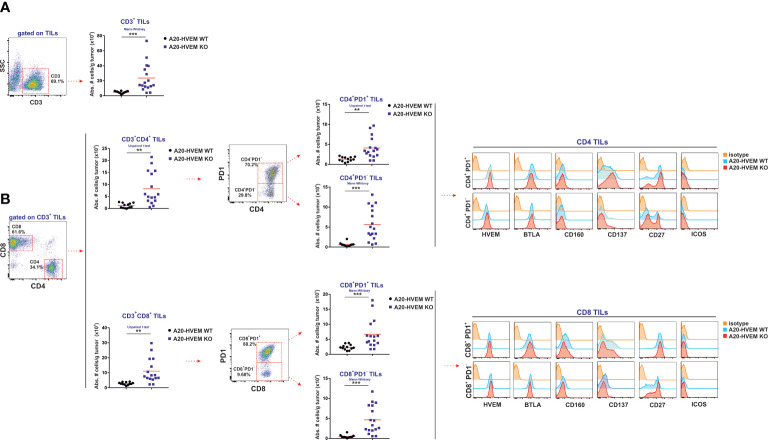
CD4 T cells and CD8 T cells with effector function are recruited to a greater extent in metastatic nodules of the liver of F1 mice receiving HVEM-deficient tumor cells than in those receiving HVEM WT tumor cells. The absolute numbers of CD3 T cells **(A)** as well as CD4 T cells and CD8 T cells tumor infiltrating lymphocytes isolated from metastatic nodules of the liver **(B)** were calculated in recipient F1 mice that received HVEM WT or HVEM-deficient A20 tumor cells. The absolute numbers of T cells PD-1^+^ and PD-1^-^ T cell subpopulations within CD4 T cells and CD8 T cells are also represented. The expression of the following activation/effector function-related markers: HVEM, BTLA, CD160, CD137, CD27 and ICOS were monitored in CD4 T cells (PD1^+^ vs PD1^-^) and CD8 T cells (PD1^+^ vs PD1^-^) infiltrating tumors. Representative dot plots depicting the gating strategy and the subpopulations of each analysis are depicted. Data show the mean from 12-16 mice per group pooled together from two independent experiments. Statistical analyses were calculated using two-tailed non-parametric Mann–Whitney U test or two-tailed unpaired *t* test. **, p<0.005 and ***, p<0.0005.

Tumor infiltrating T cells were quantified in the metastatic nodules of the liver to monitor the ongoing immune attack. The absolute number of CD3 T cells infiltrating tumors arisen from HVEM deficient leukemia cells was statistically significantly greater than that originated from HVEM WT leukemia cells (**Figure 4A**). This enhanced CD3^+^ T cell infiltration in HVEM KO tumors compared to HVEM WT tumors signified a 5-fold increase that had an impact on the expansion of both CD4 T cells and CD8 T cells, that augmented approximately 6-fold and 4-fold, respectively (**Figure 4B**). Each of the T cell subpopulations were then split up into PD-1^+^ and PD-1^-^ T cells. These T cell populations were also significantly increased in both tumor infiltrating CD4 T cells and CD8 T cells, regardless of whether they were positive or negative for PD-1. However, the magnitude of the increase was greater for the PD-1^-^ T cell subset than for the PD-1^+^ T cell subset. Thus, a 10-fold increase of CD4^+^/PD-1^-^ T cells and CD8^+^/PD-1^-^ T cells was recorded in HVEM KO tumor versus HVEM WT tumors, which contrasted with only 3-fold increase in the PD-1^+^ T cell population for both CD4 T cells and CD8 T cells. Therefore, it is likely that despite both PD-1^+^ and PD-1^-^ T cells are collaborating in their effort to resist tumor progression, PD-1^-^ T cell population contribution to the overall resistance is likely to be more important than the prone to exhausted or exhausted PD-1^+^ T cell population in their joined endeavor to resist tumor progression and dissemination.

To dissect the degree of T cell activation within the tumor, immune infiltrates in HVEM WT and HVEM KO tumors isolated from liver metastases were stained with a set of antibodies against molecules of the HVEM/BTLA/CD160 pathway as well as CD27, ICOS and CD137. No changes in expression were seen for HVEM or BTLA on either CD4 T cells or CD8 T cells that were positive or negative for PD-1 (**Figures 4A, B**, upper and lower panel). However, upregulation of CD137 was observed to be stronger in CD4 T cells infiltrating HVEM deficient tumors than in HVEM WT tumors, which is suggestive of ongoing CD4 T cell activation (**Figure 4B**, histograms upper panel).

On the other hand, CD160 expression was however upregulated in CD8^+^ / PD-1^+^ T cells infiltrating both types of tumors when compared to CD8^+^ / PD-1^-^ T cells. The frequency of CD4 T cells and CD8 T cells expressing CD27 or CD137 (4-1BB) was more strongly expressed in PD-1 positive cells than in the PD-1 negative T cell fraction of the immune infiltrates in both types of tumors (**Figure 4B**, histograms lower panel).

In summary, PD-1^-^ progenitors of effector cells are likely to be more relevant to tumor rejection than the more differentiated exhausted PD-1^+^ progeny within the metastatic immune infiltrates.

### Significant enhanced tumor immune infiltrates of short-lived effector cells (SLECs) and memory precursors of effector CD8 T cells (MPECs) in HVEM deficient tumors

To get a deep understanding on the T cell heterogeneity and the diversity of T cells infiltrating the tumors and evaluate the course of T cell differentiation, we then went on assessing the number of effector cells infiltrating the nodules of the liver metastases. IL-7Rα and KLRG-1 surface markers were used to differentiate the two stages of T cell differentiation at the effector phase of the anti-tumor response: short-lived effector cells (SLECs) and memory precursor effector cells (MPECs) (13, 45, 46). In a resolving antigenic response such as an acute infection, the two populations of responding T cells coexist. Short-lived effector cells (SLECs) clonally expand and reach a plateau at the peak of the response. After that, T cells undergo an attrition process declining soon after antigen has been cleared, leaving behind a minor subset of memory precursor effector cells (MPECs) that remains long-term after the contraction phase of the response to generate new waves of effector cells upon re-stimulation. On the contrary, if antigen persists, as it is the case in anti-tumor responses, the effector T cells and memory precursors of T cells may become exhausted due to their inability to wipe out tumor cells (47–50).

**Figure 5:**
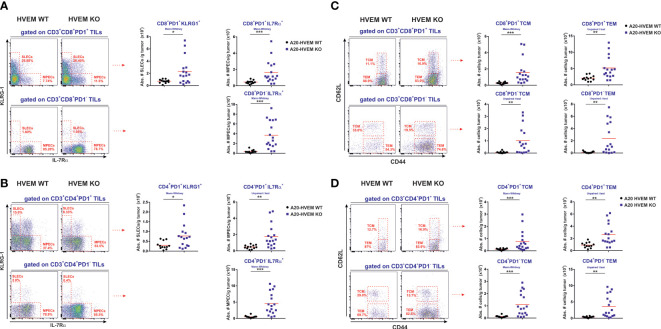
Significant enhanced infiltration of effector and memory type T cells in HVEM-deficient tumors compared to their HVEM WT counterparts. CD8^+^ PD1^+^ T cells and CD8^+^ PD1^-^ T cells as well as CD4^+^ PD1^+^ T cells and CD4^+^ PD1^-^ T cells infiltrating the metastatic hepatic nodules were analyzed for the expression of effector and memory T cell markers (KLRG-1/IL-7Rα and CD44/CD62L). **(A, B)** The absolute numbers of CD8 T cells and CD4 T cells displaying a SLECs phenotype (KLRG-1^+^/IL-7Rα^-^) coexpressing PD1 and MPECs (KLRG-1^+^/IL-7Rα^-^) coexpressing PD1 or negative for PD-1 coexpressing IL-7Rα alone are depicted. The absolute number of PD-1^+^ and PD-1^-^ CD8 T cells **(C)** and CD4 T cells **(D)** expressing CD44 and/or CD62L was also calculated. TCM: CD44^+^/CD62L^+^ and TEM: CD44^+^/CD62L^-^. Representative dot plots depicting the gating strategy and the subpopulations of each analysis is shown. Data indicate the mean value obtained from 12-16 mice per group from two independent experiments pooled together. Statistical analyses were calculated using two-tailed non-parametric Mann–Whitney U test or two-tailed unpaired *t* test. *, p<0.05, **, p<0.005 and ***, p<0.0005.

We observed a significant increase in the number of short-lived effectors (SLECs) and memory precursors of effector CD8 T cells in the immune infiltrate of metastatic hepatic nodules originated from HVEM KO tumors compared to those arisen from HVEM WT tumors (**Figure 5A**, upper panel). The absolute number of effectors PD-1+ CD8 T cells, defined as SLECs (KLRG-1+ / IL7Rα-) and MPECs (KLRG-1- / IL-7Rα+) were significantly augmented in tumor infiltrates of HVEM KO tumors compared to that of HVEM WT tumors (**Figure 5A**, upper panel). The SLECs PD1^+^ CD8 T cell population (KLRG-1^+^ / IL-7Rα^-^) that clonally expanded in response to the tumor was restricted to CD8 T cells co-expressing PD-1, as SLECs PD-1^-^ CD8 T cells were almost undetectable in the tumor infiltrates of both HVEM WT and HVEM KO tumors (**Figure 5A**, lower panel). The population of PD-1^-^ / KLRG-1^-^ / IL-7Rα^+^ cells may correspond to a bystander naïve CD8 T cell population migrating to the tumor attracted by the inflammatory environment of the tumor. Alternatively, it may represent a stem-cell like progenitor within the early effectors that contributes to the generation of new waves of effector cells produced continuously to counteract tumor growth (**Figure 5A**, lower panel).

A parallel T cell differentiation process was seen similar to that of CD8 T cells when CD4 T cells were classified and analyzed based on PD-1 and KLRG-1 / IL-7Rα expression. Both PD-1 positive SLEC and MPECs T cells were significantly augmented in HVEM deficient tumors compared to HVEM WT counterparts (**Figure 5B**, upper panel). Within the PD-1 negative, a major subpopulation of CD4 T cells expressing IL-7Rα and negative for KLRG-1 was found in greater number in HVEM KO tumors than in HVEM WT tumors, whose phenotype was compatible with an early effector with stem cell-like properties (**Figure 5B**, lower panel). To the global increase of tumor immune infiltrates, PD-1^-^ MPECs population accounted for about 10-fold expansion, whereas the PD-1^+^ MPEC population only represented a 4-fold increase when immune infiltrates in HVEM KO tumors were compared to HVEM WT tumors.

In summary, PD-1^-^ MPECs are likely to be the major contributors to the control of tumor growth in F1 recipients of HVEM KO tumors compared to HVEM WT tumors.

### More abundant central memory and effector memory T cell infiltration in HVEM deficient tumors than in HVEM WT tumors

As mentioned before, CD8 T cells responding to tumors are a heterogeneous population with different subsets of effector cells and memory cells at different stages of T cell differentiation. MPECs represent the precursors of the memory pool that in turn give rise to the two major memory T cell subpopulations. In mice, a practical classification of T cell populations displaying effector/memory phenotype is based on high expression of CD44. This permits the distinction of central memory T cells (TCM, CD44^+^ CD62L^+^) that resides in the secondary lymphoid organs with the greatest proliferative activity among the memory T cell subsets that rapidly expand and differentiate upon rechallenge and effector memory T cells (TEM, CD44^+^ CD62L^-^) that traffic between blood and the secondary lymphoid organs (51). Upon re-stimulation, central memory T cells exhibit potent proliferative responses and can generate new waves of effector cells in the secondary lymphoid organs and new effector memory T (TEM) cells with more immediate effector function, although limited lifespan and weaker proliferative potential capable to trafficking between SLO and periphery (52).

Following this general scheme, PD-1^+^ CD8 T cells exhibiting phenotype of TCM (CD44^+^ CD62L^+^) or TEM (CD44^+^ CD62L^-^) populations infiltrating HVEM WT tumors and HVEM KO tumors were evaluated. TCM CD8 T cells co-expressing PD-1^+^ and displaying an effector memory phenotype CD44^+^ / CD62L^+^ that usually home and reside within regional lymph nodes were also found significantly more increased in tumor infiltrates originated from HVEM KO leukemia cells than in tumors originated from HVEM WT leukemia cells (**Figure 5C**). The population of memory effector T cells expressing CD44^+^ and negative for CD62L (TEM) was also significantly more numerous in metastatic tumors stemming from HVEM deficient leukemia cells than in tumors originated from HVEM WT leukemia cells (**Figure 5C**). Similarly, a population of CD8/CD44 double positive T cells of TCM and TEM phenotype was also found significantly more abundant within the PD-1 negative fraction of CD8 T cells infiltrating HVEM KO tumors than in HVEM WT tumors. However, the level of CD44 expression in PD-1^-^ CD8 T cells was intermediate/low when compared to that observed in PD-1^+^ CD8 T cells (**Figure 5C**).

Following a similar analytical approach, a population of CD4 T cells either positive or negative for PD-1 displaying a TCM or TEM phenotype was also found significantly increased in immune infiltrates of HVEM KO tumors when compared to HVEM WT tumors (**Figure 5D**). Once again, the level of CD44 expression was reduced in the PD-1 negative population compared to the PD-1 positive population (**Figure 5D**). This population negative for PD-1 within CD4 T cells and CD8 T cells exhibiting a TEM phenotype may represent a stem-cell like progenitor that after T cell activation remains active to generate new waves of effector T cells in response to the demand of a constant presence of tumor antigen.

In summary, the magnitude of the preferential rise of the PD-1^-^ TCM and TEM subpopulations of both CD4 T cells and CD8 T cells represented about 10-11-fold expansion, whereas the PD-1^+^ TCM population augmented 6-fold and the PD-1^+^ TEM population only expanded 2-3 times when immune infiltrates in HVEM KO tumors were compared to HVEM WT tumors.

Overall, despite the diversity and heterogeneity of the PD-1^+^ and PD-1^-^ populations within CD4 T cells and CD8 T cells expressing effector/memory phenotypic markers present in the immune infiltrates, the PD-1^-^ of population of TCM and TEM T cells was by far more abundantly present in HVEM KO tumors than in HVEM WT tumors.

### Significant expansion of an early effector with stem cell-like progenitor properties (PD-1^-^ / Tim3^-^) CD8 T cells and CD4 T cells in immune infiltrates of tumors originated from HVEM deficient leukemia cells

Anderson et al., proposed that both PD-1^-^ and PD-1^+^ subpopulations of CD8 T cells infiltrating tumors are essential for the control of tumor growth. As a summary of their observations, they put forward the linear T cell differentiation model described in **Supplementary Figure 1** of the material and methods section (27, 28).

**Figure 6:**
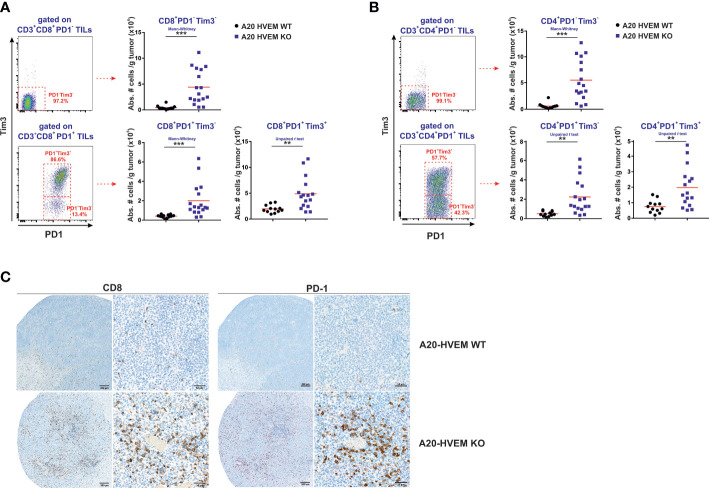
All three stages of the linear T cell differentiation model were significantly augmented in F1 recipients receiving HVEM deficient tumor cells. Hepatic metastatic nodules from F1 mice receiving HVEM WT or HVEM KO tumor cells were collected and the absolute number of CD8 T cells **(A)** and CD4 T cells **(B)** of stem cell like effector phenotype (PD1^-/^Tim3^-^), exhausted T cells (PD1^+^/Tim3^-^) and terminally differentiated (PD1^+/^Tim3^+^) cells was calculated. Representative dot plots depicting the gating strategy and the subpopulations of each analysis is shown. Data illustrates the mean from 12-16 mice per group from two independent experiments. Statistical analyses were calculated using two-tailed non-parametric Mann–Whitney U test or two-tailed unpaired *t* test. **(C)** Immunoperoxidase labeling of paraffin-embedded sections was performed with anti-CD8 or anti-PD-1 antibodies in metastatic hepatic lesions of F1 recipients engrafted with either A20 HVEM WT or A20 HVEM KO tumor cells. Microscopic photos were obtained with an objective 20X and then the regions of interest were digitally magnified and represented with a bar and its equivalence in microns. **, p<0.005 and ***, p<0.0005.

In line with this report, we have identified a major population of PD-1^-^ / Tim 3^-^ within CD8 T cells and also within CD4 T cells that infiltrated significantly better HVEM deficient tumors than HVEM WT tumors (**Figures 6A, B**, upper panel). Either CD8 T cells or CD4 T cells displayed features compatible with an early effector population positive for IL-7Rα that exhibited a considerable expression of CD44, although the fluorescence intensity was inferior to that observed in PD-1^+^ T cells (**Figures 5C, D**). Remarkably, all these three stages of the linear T cell differentiation model were significant augmented not exclusively in CD8 T cells but also found to be shared by CD4 T cells (**Figures 6A, B**, upper panel).

The extent of the expansion of the PD-1^-^/Tim3^-^ stem cell like population represented a 10-fold increase in both CD4 T cells and CD8 T cells, whereas PD-1^+^/Tim3^-^ (precursor of exhausted T cells) and PD-1^+^/Tim3^+^ (terminally exhausted T cells) only increased 4-5-fold and 2.5-fold, respectively.

All in all, the magnitude of the expansion of the stem cell-like PD-1^-^ /Tim3^-^ population was more prominent than that of precursors of exhausted T cells (PD-1^+^/Tim3^-^) and terminally exhausted T cells (PD-1^+^/Tim3^+^) in HVEM deficient tumors versus HVEM WT tumors.

### T cells co-expressing PD-1 and/or Tim 3 were significantly boosted in immune infiltrates of HVEM deficient tumors

Once T cells reached the effector phase of the response, which occurs about 7-12 days after the initial exposure to antigen, if antigen persists chronically, like in anti-tumor responses, the contraction phase of the response never ensues. In this chronic scenario of antigen persistence, the pool of memory T cells undergoes epigenetic changes that leads to the gradual and progressive conversion of T cell precursors of exhausted T cells (TpEX) into distinct transitional stages of exhausted T cells (Tex) that end up in a population of terminally differentiated exhausted T cells. The latter, shows progressive and hierarchical loss of effector cytotoxic functions, diminished proliferation and sustained upregulation and co-expression of multiple co-inhibitory receptors (28, 47, 49, 50, 53). This conversion from precursors of exhausted T cells to terminal exhausted T cells goes hand in hand with the upregulation of Tim 3 expression that serves for its phenotypic characterization (28, 54). Our results confirmed that all three stages of the linear differentiation T cell model described in **Supplementary Figure 1** were present in both CD8 T cells and CD4 T cells, being Tim-3 expression restricted to PD-1^+^ T cells, whereas was completely absent in the PD-1 negative pool of T cells.

As mentioned in the previous paragraph, the early effectors PD-1^-^ / Tim 3^-^ are the source of precursors of exhausted T cells (TpEx), which are PD-1^+^ / Tim 3^-^. This population of PD-1^+^ / Tim 3^-^ CD8 T cells and PD-1^+^ / Tim 3^-^ CD4 T cells was significantly expanded in the immune infiltrates of HVEM deficient tumors compared to HVEM WT tumors (**Figures 6A, B**). Interestingly, the more differentiated population of PD-1^+^ / Tim-3^+^ previously classified as terminally exhausted T cells was also significantly augmented in the immune infiltrates of HVEM deficient tumors compared to that of HVEM WT tumors (**Figures 6A, B**). Altogether, these findings suggest that expression of Tim-3 might not be necessarily associated with a dysfunctional state of unresponsiveness and is likely to be linked to a transitional stage of exhaustion in which the functional activity of T cells is still conserved and reflects effective effector function.

Extensive perivascular accumulation of CD8 T cells and PD-1^+^ T cells were found in tissues sections of metastatic hepatic lesions of tumors arisen from HVEM deficient tumors compared to those originated from HVEM KO tumors (**Figure 6C**)

In conclusion, despite the fact that the major T cell population infiltrating HVEM deficient tumors is negative for PD-1 expression, the two PD-1^+^ T cell populations represented by the precursors of exhausted T cells and the terminally exhausted T cells may also join forces to tackle tumor progression despite the limited functional activity of the latter.

### Downregulation of Ly108 (CD352, Slamf6) and TCF-1 transcription factor expression occurs as T cells progress in the course of T cell differentiation towards terminally exhausted T cells concurrently with accumulation of co-inhibitory receptors

**Figure 7:**
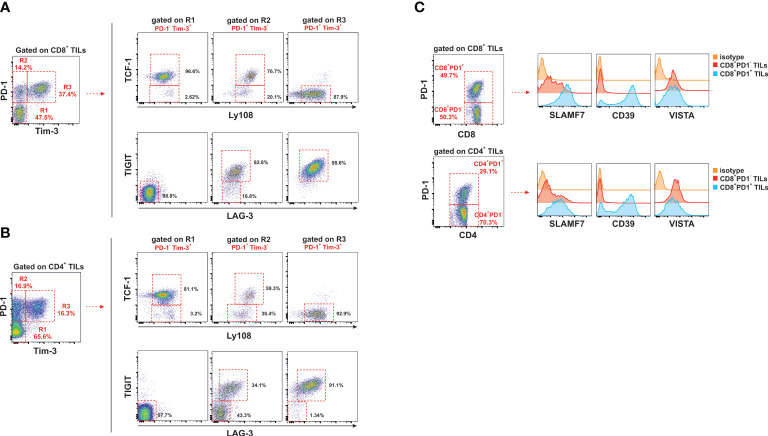
Chronic antigen persistence despite an ongoing immune response leads to the formation of terminally exhausted T cells. Accumulation of co-inhibitory receptors as T cells advanced in the course of T cell differentiation towards terminally differentiated exhausted T cells goes hand by hand with downregulation of TCF-1 transcription factor and Ly108 surface marker (Slamf6). CD8 T cells and CD4 T cells were gated into three regions on the basis of PD-1 and Tim 3 expression: R1 (PD-1^-^/Tim 3^-^, stem cell like T cells), R2 (PD-1^+^/Tim 3^-^, precursors of exhausted T cells) and R3 (PD-1^+^/Tim 3^+^, exhausted T cells). The expression of the transcription factor TCF-1 and the surrogate surface marker Ly108 (Slamf6) was evaluated in these three subpopulations **(A)** along with the expression of co-inhibitory receptors LAG-3 and TIGIT **(B)**. **(C)** Upregulation of Slamf7 and CD39 expression on CD4 T cells and CD8 T cells expressing PD-1. No changes in VISTA expression were seen. The plots are from one experiment representative out of two performed.

*Tcf7* gene encodes the transcription factor TCF1 whose expression alone or in combination with the surrogate cell membrane molecule Ly108 permits a clear distinction of the three typical stages of the T cell differentiation linear model found in chronic infections and cancer (55, 56). Moreover, the expression of TCF1 seems to play a critical role in the development and/or maintenance of the stem cell-like early effectors, which are the source of progenitors of new waves of effectors and memory T cells during the response to chronic stimuli. Under conditions of antigen persistence, T cells infiltrating tumors accumulate co-inhibitory receptors as they progress in the course of T cell differentiation towards terminally exhausted T cells (57). The increase in expression of TCF1 and Ly108 as well as co-inhibitory receptors was monitored along the course of the T cell differentiation process in T cell subpopulations based on the combined PD-1 and Tim 3 expression. As T cell differentiation progresses, TCF1 intracellular transcription marker and the surrogate cell membrane Ly108 gradually diminished their expression in both CD4 T cells and CD8 T cells (**Figures 7A, B**, upper panel). This was accompanied with a progressive increase in coexpression of LAG-3 and TIGIT as well as upregulation of Slamf7 and CD39 molecules (**Figures 7A, B**, lower panel, **Figure 7C**). However, no substantial changes in VISTA (PD-1H homologous) expression were found.

On the contrary, the expression or overexpression of HVEM gives a survival advantage to tumors that often correlates with poor prognosis and increased tumor aggressiveness in many solid tumors (58–65). Gradually, these observations have been extended to tumors of hematopoietic origin (66–72). It seems that the deficiency caused by the lack of HVEM expression and the subsequent inability to co-inhibit T cell responses through BTLA provides the immune system with a competitive advantage to curb tumor growth. This is reflected by a more intense PD-1^+^ / Tim 3^-^, and PD-1^+^ / Tim 3^+^ T cell infiltration in tumors derived from HVEM deficient cells than in those originated from HVEM WT tumor cells. The significant increased presence of stem cell-like early effector PD-1^−^ T cells in tumors lacking HVEM guarantees the seeding of a continuous source of new waves of effector T cells that can mature to memory type T cells with different degrees of immediate effector functional activity to maintain long-term anti-tumor immune responses.

Altogether these findings indicated that primarily effectors PD-1^-^ and on a second level of importance memory type PD-1^+^ T cells are active participants of immune infiltrates controlling tumor growth in HVEM deficient tumors, which are unable to co-inhibit T cell responses. This highlights the importance of HVEM as a ligand for the co-inhibitory receptor BTLA in the modulation of the anti-tumor responses.

## Discussion

In this work, a CRISPR/Cas9 strategy was adopted to abrogate HVEM expression in A20 leukemia cells. The data presented herein indicates that HVEM behaves predominantly as a co-inhibitory receptor since the genetic inactivation of HVEM expression on tumor cells enhances T cell infiltration and improves tumor control. These findings are in line with the preferential co-inhibitory function of HVEM observed in HVEM and BTLA deficient mice, which are more susceptible to undergo experimentally-induced autoimmune disease (1, 7). HVEM endowed leukemia cells with a survival advantage and protected them from immune attack facilitating tumor dissemination and the formation of tumor metastases in the hematopoietic and the non-hematopoietic compartments despite the existence of natural barriers of resistance (NK cells and CD8 T cells) to tumor implantation in F1 recipients.

The parental engraftment of tumor cells into F1 recipients offers a set of interesting features as a preclinical model from an immunological perspective. One of them is that both NK cells and CD8 T cells work together to resist parental tumor engraftment in F1 recipients (23). Besides, the endogenous tumor specific antigens naturally arisen by somatic mutation along with those introduced by genetic manipulation of the cell line are cross-primed by the indirect pathway of antigen presentation stimulating CD8 T cell and CD4 T cell cytotoxic activity. In this sense, A20 leukemia cell line is deemed to be a good preclinical model since it bears an intrinsic high tumor mutational burden and consequently behaves as highly immunogenic. This antigenic tumor burden correlates quite well with effective responses in preclinical animal models and in humans, which makes the tumor model amenable for immune checkpoint blockade intervention and preclinical evaluation (40).

Somatic HVEM mutations are often present in a large variety of hematopoietic tumors, such as FL and DLBCL (67–69). Recurrent deletions of HVEM have also been observed in classical Hodgkin lymphoma (70). Mutations affecting the binding site of HVEM interaction with BTLA in *cis* promotes B cell lymphoma development because of unleashed BCR signaling and dysregulation of B cell activation and further differentiation (71). A high number of follicular B cell lymphomas accumulate mutations in the HVEM gene, some of which may lead to the loss of HVEM functional activity and consequently the impossibility to co-inhibit follicular T cell help through *trans* interaction with BTLA. This absence of HVEM/BTLA *trans* signal can function as a driver mutation in lymphomagenesis because of excessive T cell help and dysregulated B cell responses in the germinal center (72, 73). Although a priori paradoxical, apart from promoting tumor development, loss of function mutations in HVEM gene also worsen tumor fitness and unleash the anti-tumor response despite the fact that both co-stimulatory and co-inhibitory signals are abrogated. The loss of HVEM expression may prevent co-inhibition of T cells upon engagement of BTLA on responding T cells and abrogate co-stimulation through CD160 and LIGHT. As co-inhibition seems to be the predominant function of HVEM over costimulation, the expectation is that tumor cells devoid of HVEM would become more vulnerable to immune attack (7). This claim is in line with our own findings since the inactivation of HVEM in A20 leukemia cells makes them more vulnerable to immune attack and hampers their implantation in spleen and bone marrow. Besides, the metastatic nodules of the liver in HVEM KO tumors were smaller and abundantly infiltrated with innate and adaptive immune cells compared with tumors originated from HVEM WT cells, although complete remission of tumor growth was not achieved. On the contrary, overexpression of HVEM gives a survival advantage to tumors that often correlates with poor prognosis and increased tumor aggressiveness in many human gastrointestinal cancers (58–62), carcinomas (63–65) and also in some hematological tumors (66).

The significant preferential increase of NK cells and to less extent NKT cells and monocytes in HVEM KO tumors compared to HVEM WT tumors may account for the early resistance to tumor engraftment. Their function however may fade away overtime as tumor cells evolve in the host and deploy evasion mechanisms to escape the immunosurveillance of the host. The significant increase of different populations of T cells in HVEM-deficient tumors was associated with a smaller size of the liver tumor metastases and limited dissemination of tumor cells to host spleen and bone marrow. The adaptive immune response was phenotypically characterized in the metastatic lesions of the liver and followed a linear scheme of T cell differentiation similar to that put forward by Anderson et al. (27, 28). First, we identified a population of stem cell-like early effectors characterized by the lack of expression of PD-1 and Tim 3 that were all IL-7Rα^+^ CD44^low/interm^ CD62L^-^, which are likely to be the direct progenitors of SLECs and MPECs. This finding indicates that PD-1 negative T cell population is not just a bystander passenger attracted by the inflamed tumor microenvironment and the cytokines and chemokines released during the course of the anti-tumor response, but otherwise a potential active contributor to the anti-tumor immune response. As antigen persists in tumors long-term despite an active anti-tumor response of the host, MPECs initiate a transcriptional program to originate precursors of exhausted T cells (PD-1^+^ / Tim 3^-^), which were CD44^high^ CD62L^-^ (74–77). From this T cell differentiation turning point through a poorly characterized multistep transitional process, a population of terminally differentiated exhausted T cells (PD-1^+^ / Tim 3^+^) would arise. This population would be more prone to enter into a dysfunctional program with gradual, progressive and hierarchical loss of effector cytotoxic functions and sustained upregulation and co-expression of multiple co-inhibitory receptors to minimize tissue damage and prevent pathology. Despite that, terminally exhausted T cells may not be as dysfunctional as previously anticipated due to their significant presence in HVEM deficient tumors compared to HVEM WT tumors and instead they may still conserved some functional activity contributing to some extent to limit tumor growth (47, 53).

Although the relative functional contribution of each T cell subset to the observed overall effect cannot be established, we in line with others favor the notion that PD-1^-^ T cell subset may be deemed a more decisive player in resisting tumor progression than the PD-1^+^ T cell subset. PD-1 negative T cells displaying stem cell-like phenotypic features would act as a reservoir of progenitors of effector cells able to replenish the compartment of effector T cells and, therefore, be responsible for the maintenance of an ongoing anti-tumor response (28). This response would persist overtime despite the loss of function of the more differentiated T cells that become exhausted in the face of a chronic presence of antigen (tumor cells), which are unable to clear tumor cells, but try to tackle their progression and subsequent dissemination.

For all the reasons above mentioned, we support the notion of caution when it comes to ascribing a dysfunctional phenotype to these T cells based on the expression of co-inhibitory receptors alone as these molecules are also found on effector T cells that retain functional properties (28, 47, 50, 78). Moreover, the tumor persistence despite the intense T cell infiltration in HVEM deficient tumors may reflect the ability of BTLA still present on tumor cells to engage HVEM expression on stromal cells to condition the tumor microenvironment and therefore override the function of tumor infiltrating immune cells.

One remarkable finding that caught our attention was the fact that CD4 T cells showed a parallel pattern of T cell differentiation similar to that of CD8 T cells going through the same three stages of the linear T cell differentiation pathway. These CD4 T cell subpopulations were also significantly increased in the metastatic lesions of the livers originated from HVEM deficient leukemia cells compared to that of HVEM WT tumors. This suggests that both T cells are similarly involved in dealing with the persistence of the tumor while they are cooperating to achieve the global control of tumor growth.

In conclusion, the set of evidences presented herein supports the postulate that in the absence of HVEM, tumors become susceptible to immune attack. This increased vulnerability is likely to be due to the impossibility of HVEM to engage BTLA in *trans* to co-inhibit T cell function. Besides, the lack of HVEM in tumors prevents cell intrinsic HVEM/BTLA *cis* interactions and BTLA become freely available to engage HVEM in stromal cells that would condition the tumor microenvironment. The combined action of all these mechanisms working together in consonance would account for the shrinking of tumor metastases in F1 mice receiving HVEM deficient tumor cells.

Ongoing research is underway to overcome some limitations of the present work in which a detailed study in immunocompetent mice was performed uniquely for a single HVEM deficient clone. Another limitation of the study was the low expression of HVEM detected in A20 cell line, although it was the only transplantable mouse tumor cell line tested in which HVEM protein could be revealed on the cell surface. Even though it may result puzzling, the commercially available antibodies against mouse HVEM only recognize the expression of HVEM in primary mouse hematopoietic cells, such as T cells and B cells and other immune cells (6, 79). As a matter of fact, mouse HVEM expression was undetectable in non-hematopoietic tumor cells lines, such as MC-38 or B16.F10 tumor cells, which was in contrast with the detectable levels of mRNA expression for mouse HVEM by RT-PCR. As opposed to mouse, human cell lines of similar phenotype display detectable surface expression of HVEM (80). To account for these differences, there may be post-translational modifications in the mouse HVEM protein expression in different cell types that may affect its recognition by currently available anti-mouse HVEM monoclonal antibodies.

The development of biologics targeting HVEM/BTLA/CD160/LIGHT is still in its infancy and will require a deeper understanding on how this pathway works during the process of T cell activation and differentiation and in the interplay tumor, tumor microenvironment, and the anti-tumor immune response. It will also be necessary to elucidate how the pathway operates in a cancer-specific manner in order to redirect and potentiate the body immune surveillance mechanisms against tumors to achieve effective anti-tumor responses.

## Data availability statement

The datum generated in this study referred as the HVEM mutation in A20 leukemia cell line is publicly available in Genbank under accession number ON646268.

## Ethics statement

All animal experiments have been approved by the Animal Welfare Committee of the University of Alcala de Henares (Madrid) and handled in accordance with the European Guidelines for Animal Care and Use of Laboratory Animals (authorization # OH-UAH-2016/015).

## Author contributions

J-IRB and M-LR designed the study, performed and analyzed the experiments and wrote the manuscript. CY-DJ initiated the project in the context of her PhD thesis and started the initial characterization of the tumor cell lines. RÁ-E provided advice on the statistical analysis of the data. GR and EC helped with their expertise in the pathological examination of the tumor samples and staining of the metastatic lesions of the liver. JAP-S contributed with reagents and their expertise in the field. All authors contributed to the final version of the manuscript.

## Supplementary material

Supplementary Figure 1 The linear model of T cell differentiation in response to the chronic persistence of antigen stimulation. In a resolving acute response, T cell activation is ensued by T cell clonal expansion of effector cells that reach a plateau to clear the triggering stimulus. Then, they undergo the contraction phase of the immune response ensues, a process of effector T cell attrition (activation-induced cell death), in which the vast majority of terminally differentiated effector T cells (SLECs) die by apoptosis, sparing a population of MPECs that gives rise to long-lived memory T cells (46, 81). Anderson et al., have put forward a linear model of T cell differentiation in response to the chronic persistence of antigen despite an ongoing immune response. If antigen persists as in tumors and cannot be cleared by the immune response, a stem like population of effector T cells (PD-1^-^/Tim 3^-^) arises that gives rise to constant new waves of short lived effector cells (SLECs) and memory precursor of effector cells (MPECs). MPECs instead of converting into long-lived memory T cells will initiate a transcriptional program of exhaustion to become precursors of exhausted T cells (PD-1^+^ / Tim 3^-^), which are CD44^high^ CD62L^-^ and can potentially be reprogrammed upon PD-1 / PD-L1 blockade. From this T cell differentiation turning point, a population of terminally differentiated exhausted T cells (PD-1^+^ / Tim 3^+^) would arise with decreased functional activity, downregulation of TCF1 transcription factor and the surrogate cell membrane molecule Ly108 (Slamf6) and co-expressing multiple co-inhibitory receptors (27, 28, 82).

Supplementary Figure 2 Generation of a HVEM-deficient A20-GFP tumor cell line using a CRISPR-Cas9 approach. **(A)** Mouse HVEM gene is encoded by eight exons. Deletion of exon 1 encoding the first part of the signal peptide led to abrogation of HVEM expression on the cell membrane of tumor cells. Different domains of HVEM are abbreviated as follows: TM, transmembrane region; CRD, Cystein-Rich Domain; IC, intracellular domain. **(B)** Cleavage efficiency of different sgRNA guides designed over exon 1: control HVEM DNA target sequence, HVEM DNA target sequence plus Cas9 or HVEM DNA plus Cas9 incubated with different sgRNA guides were loaded and run in a 1% agarose gel. **(C)** PCR amplification of HVEM mutation of exon 1 from A20-GFP HVEM WT (LCL-7) tumor cell line and A20-GFP HVEM KO deficient tumor cell line (LCL-25). The expected band for the amplicon obtained with the flanking primers designed on exon 1 was 1090 bp (A20 HVEM WT), whereas in HVEM deficient tumor cell line was 618 bp. The indel mutation introduced into exon 1 encompassed 472 bp affecting the 5´UTR region, exon 1, exon 1 and part of intron 1-2 sequences. **(D)** Amino acid sequence alignment showing the complete deletion of the first part of the signal peptide encoded by exon 1 of mouse HVEM gene. The alignment of the amino acid sequence of the HVEM WT protein versus HVEM KO protein was performed with Clustal Omega (http://www.ebi.ac.uk/tools/clustalo/. An asterisk displays identical amino acids indicating perfect alignment (*). **(E)** Flow cytometry histograms of A20 HVEM WT, A20-GFP HVEM WT (LCL-7) and A20-GFP HVEM KO (LCL-25) cell lines stained with biotinylated isotype matched control (rat IgG2a, AFRC MAC 157) antibody or biotinylated rat anti-mouse HVEM monoclonal antibody (clone 6C9) and biotinylated rat anti-mouse BTLA (clon 4G12b) mAbs. The binding reactions were developed using streptavidin (SA)-Alexa Fluor 647 or SA-BV421. **(F)** *In vitro* rate of proliferation of HVEM WT and HVEM KO cell lines. Four replicates of A20 HVEM WT or A20 HVEM KO tumor cells were seeded in a 24 well plate at the rate of 5000 cells per well and cell counting was performed every day over a period of 6 days. Total number of cells (×10^6^) in culture is plotted at different time points.

## Glossary

**Table:**

CD	Cluster of differentiation
NK	Natural killer
NKT	Natural killer T cells
TCR	T cell receptor
MHC	Major histocompatibility complex
WT	Wild type
KO	Knock-out
APC	Antigen presenting cells
MFI	Mean fluorescence intensity
BMCs	Bone marrow cells
mAb	Monoclonal antibody
PI	Propidium Iodide
FCS	Fetal calf serum
SD	Standard deviation
F1 (Balb/c × B6)	Balb/c (female) x B6 (male) F1 hybrid
IFN-γ	gamma interferon
CRISPR	Clustered regularly interspaced short palindromic repeats
i.p.	intraperitoneal
PBS	Phosphate buffer saline. ILC, Innate lymphoid cells
SFM	Serum free medium. HVEM, Herpesvirus entry mediator
BTLA	B-and T-lymphocyte attenuator
LIGHT	homologous to lymphotoxin, exhibits inducible expression and competes with HSV glycoprotein D for binding to herpesvirus entry mediator, a receptor expressed on T lymphocytes
LTα	Lymphotoxin alpha
PD-1	Programmed cell death –1
PD-L1	PD-1 ligand
TNFSF	Tumor necrosis factor superfamily
TNFRSF	Tumor necrosis factor receptor superfamily
CRD	Cysteine rich domains
PCR	Polymerase chain reaction
DLBCL	Diffuse Large B Cell Lymphoma
TEM	T effector memory
TCM	T central memory
MPECs	Memory precursor effector cells
SLECs	Short lived effector cells
Tim–3	T-cell immunoglobulin and mucin-domain containing-3
GFP	Green fluorescence protein
TILs	tumor infiltrating leukocytes
TM	Transmembrane
IC	Intracellular
FL	Follicular lymphoma
DLBCL	Diffuse large B cell lymphoma
IL-7Rα	Interleukin receptor 7 alpha chain
SA	Streptavidin
AF	Alexa fluor
DNA	deoxyribonucleic acid
RNA	Ribonucleic acid
FSC	Forward scatter
SSC	Side scatter
TCF-1	T cell factor 1
sgRNA	single guide RNA
