# Supplementary figures and images for: Genetic deletion of HVEM in a leukemia B cell line promotes a preferential increase of PD-1- stem cell-like T cells over PD-1+ T cells curbing tumor progression
Source: Front Immunol. 2023 Mar 23;14:1113858. doi: 10.3389/fimmu.2023.1113858 (PMC10076739; doi:10.3389/fimmu.2023.1113858)

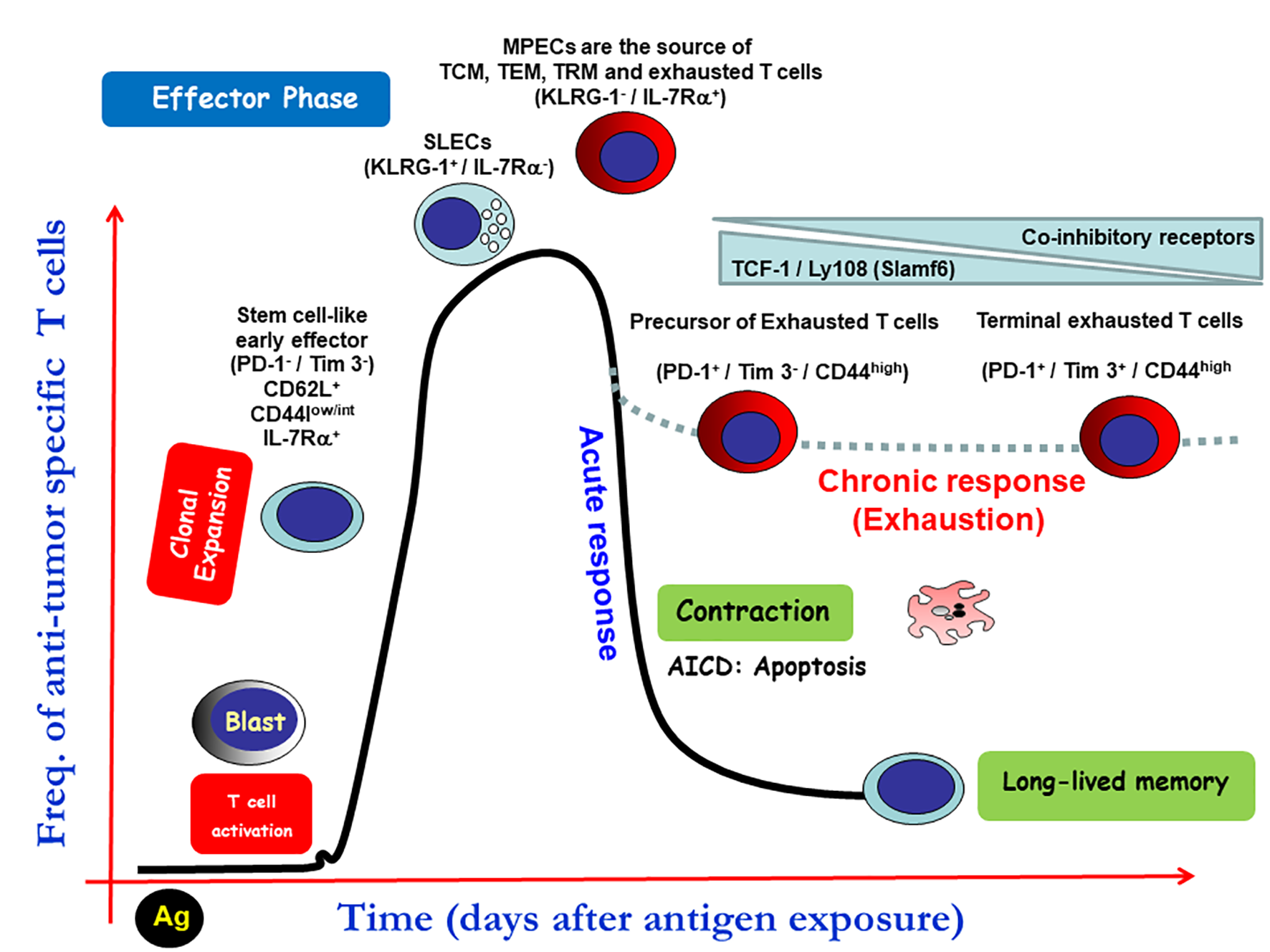

Supplement: Supplementary Figure 1 — The linear model of T cell differentiation in response to the chronic persistence of antigen stimulation. In a resolving acute response, T cell activation is ensued by T cell clonal expansion of effector cells that reach a plateau to clear the triggering stimulus. Then, they undergo the contraction phase of the immune response ensues, a process of effector T cell attrition (activation-induced cell death), in which the vast majority of terminally differentiated effector T cells (SLECs) die by apoptosis, sparing a population of MPECs that gives rise to long-lived memory T cells (46, 81). Anderson et al., have put forward a linear model of T cell differentiation in response to the chronic persistence of antigen despite an ongoing immune response. If antigen persists as in tumors and cannot be cleared by the immune response, a stem like population of effector T cells (PD-1-/Tim 3-) arises that gives rise to constant new waves of short lived effector cells (SLECs) and memory precursor of effector cells (MPECs). MPECs instead of converting into long-lived memory T cells will initiate a transcriptional program of exhaustion to become precursors of exhausted T cells (PD-1+ / Tim 3-), which are CD44high CD62L- and can potentially be reprogrammed upon PD-1 / PD-L1 blockade. From this T cell differentiation turning point, a population of terminally differentiated exhausted T cells (PD-1+ / Tim 3+) would arise with decreased functional activity, downregulation of TCF1 transcription factor and the surrogate cell membrane molecule Ly108 (Slamf6) and co-expressing multiple co-inhibitory receptors (27, 28, 82). [file Image_1.tif]

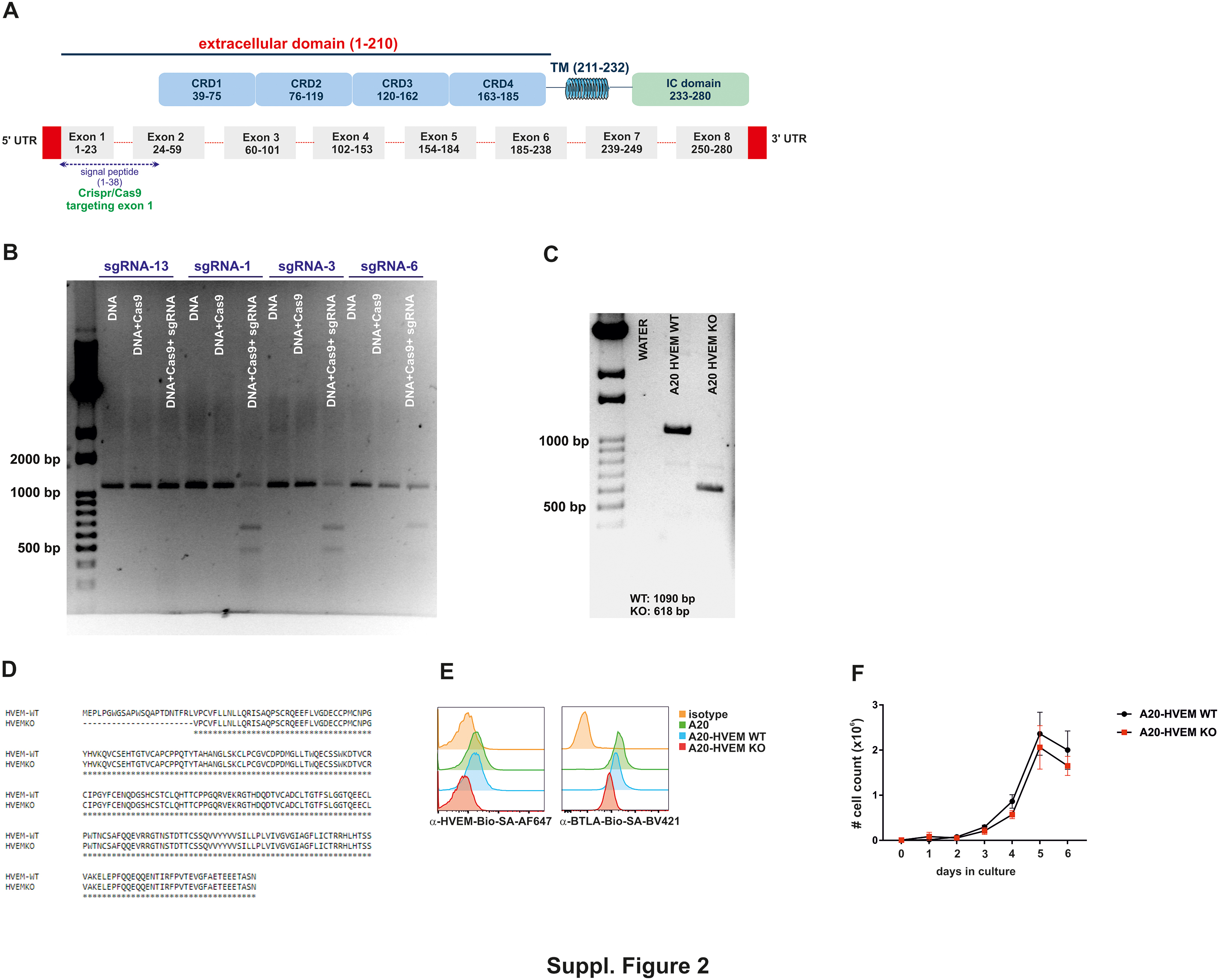

Supplement: Supplementary Figure 2 — Generation of a HVEM-deficient A20-GFP tumor cell line using a CRISPR-Cas9 approach. (A) Mouse HVEM gene is encoded by eight exons. Deletion of exon 1 encoding the first part of the signal peptide led to abrogation of HVEM expression on the cell membrane of tumor cells. Different domains of HVEM are abbreviated as follows: TM, transmembrane region; CRD, Cystein-Rich Domain; IC, intracellular domain. (B) Cleavage efficiency of different sgRNA guides designed over exon 1: control HVEM DNA target sequence, HVEM DNA target sequence plus Cas9 or HVEM DNA plus Cas9 incubated with different sgRNA guides were loaded and run in a 1% agarose gel. (C) PCR amplification of HVEM mutation of exon 1 from A20-GFP HVEM WT (LCL-7) tumor cell line and A20-GFP HVEM KO deficient tumor cell line (LCL-25). The expected band for the amplicon obtained with the flanking primers designed on exon 1 was 1090 bp (A20 HVEM WT), whereas in HVEM deficient tumor cell line was 618 bp. The indel mutation introduced into exon 1 encompassed 472 bp affecting the 5´UTR region, exon 1, exon 1 and part of intron 1-2 sequences. (D) Amino acid sequence alignment showing the complete deletion of the first part of the signal peptide encoded by exon 1 of mouse HVEM gene. The alignment of the amino acid sequence of the HVEM WT protein versus HVEM KO protein was performed with Clustal Omega (http://www.ebi.ac.uk/tools/clustalo/. An asterisk displays identical amino acids indicating perfect alignment (*). (E) Flow cytometry histograms of A20 HVEM WT, A20-GFP HVEM WT (LCL-7) and A20-GFP HVEM KO (LCL-25) cell lines stained with biotinylated isotype matched control (rat IgG2a, AFRC MAC 157) antibody or biotinylated rat anti-mouse HVEM monoclonal antibody (clone 6C9) and biotinylated rat anti-mouse BTLA (clon 4G12b) mAbs. The binding reactions were developed using streptavidin (SA)-Alexa Fluor 647 or SA-BV421. (F) In vitro rate of proliferation of HVEM WT and HVEM KO cell lines. Four replicates of A20 HVEM WT or A20 HV [file Image_2.jpeg]
